# A prospective study in adolescent idiopathic scoliosis affected by thoracolumbar and lumbar curves in treatment with a progressive action short brace (PASB): assessment of results according to the SRS committee on bracing and nonoperative management standardization criteria

**DOI:** 10.1186/1748-7161-8-S2-O60

**Published:** 2013-09-18

**Authors:** Angelo Gabriele Aulisa, Vincenzo Guzzanti, Marco Giordano, Giuseppe Mastantuoni, Marco Peruzzi, Lorenzo Aulisa

**Affiliations:** 1 Orthopaedic Department, Children's Hospital Bambino Gesù, Institute of Scientific Research, Rome, Italy

## Background

The effectiveness of conservative treatment of scoliosis is controversial. In recent retrospective studies, we have demonstrated the effectiveness of PASB in correcting and holding thoracolumbar and lumbar curves. Furthermore, several papers have recently been published on adolescent idiopathic scoliosis in agreement with SRS criteria; however, none of them was prospective. 

## Purpose

The purpose of this study was to confirm the effectiveness of PASB in the correction of thoracolumbar and lumbar curves, in agreement with the SRS Committee on Bracing and Nonoperative Management Standardization Criteria in a prospective study. .

## Methods

We have carried out a prospective study of 197 adolescents (mean age 11.8 ± 0.5 years) treated from 1993 to 2012 with thoracolumbar and lumbar curve and a pre-treatment Risser scores ranging from 0 to 2. To evaluate the effectiveness of treatment and standardize the results, SRS criteria were used. All patients were prescribed a full-time PASB. The minimum duration of follow-up was 24 months. Anteroposterior radiographs were used to estimate the curve magnitude (CM) and the torsion of the apical vertebra (TA) at five time points: beginning of treatment (t1), one year after the beginning of treatment (t2), intermediate time between t1 and t4 (t3), end of weaning (t4), and 2-year minimum follow-up from t4 (t5). Three outcomes were distinguished: curve correction, curve stabilization and curve progression. Statistical analyses were performed with GraphPad Prism 6. 

## Results

Of the total patients in the study, 136 (69%) had at least two years of follow-up, 37 (19%) were still in treatment and 24 (12%) abandoned the treatment. Of the 24 patients who abandoned the treatment, , there was curve correction in 20 cases, curve stabilization in three cases and curve progression in one case at the time of abandonment. Of these, nine were revised to follow-up and one patient was recommended for surgery. In patients with a definite outcome CM mean value was 29.06 ± 4.73 SD at t1 and 13.58±8.7 SD at t5. TA was 11.56±5.53 at t1 and 7.4±4.93 at t5. The variations between measures of Cobb and Perdriolle degrees between CM t5-t1 and TA t5-t1 were statistically significantly different. Curve correction was accomplished in 120 patients (88%), whereas curve stabilization was obtained in 13 patients (10%). Three patients (2%) had a curve progression and nobody was recommended for surgery. 

## Conclusions and discussion

Our study confirmed that the PASB is highly effective in treating thoracolumbar and lumbar curves, with most patients reaching a curve correction, and is especially effective in reducing surgical rates. Moreover, the PASB has an excellent compliance, with a dropout rate of only 12%. 
